# Irisin and the therapeutic benefits of exercise

**DOI:** 10.1186/1753-6561-6-S3-O23

**Published:** 2012-06-01

**Authors:** Bruce M Spiegelman, Stanley J Korsmeyer

**Affiliations:** 1 Dana-Farber Cancer Institute, Harvard Medical School, USA

## 

We are experiencing a worldwide epidemic of obesity and type II diabetes. Our group has been interested in the development of both white and brown fat, particularly at the level of gene transcription. PGC1α was first described as a coactivator of PPARγ in the control of brown fat-mediated thermogenesis. More recent work has shown that PGC1α controls much of an exercise program in skeletal muscle. We have now found that PGC1α expression and exercise control the expression of Fndc5, a membrane protein of skeletal muscle. Fndc5 is proteolysed to give rise to a new secreted protein of 112 amino acids that we have called irisin. Irisin circulates in both muse and man, and even mild elevations of irisin activates the browning of white fat, causing increased energy expenditure and reducing obesity. Irisin administration via adenoviral vectors also greatly improves glucose homeostasis in high-fat fed mice. Most recently we have identified cell surface binding of irisin that is likely to represent a cell surface receptor. These data indicate that irisin is a protein regulated in muscle by exercise that gives some of the benefits of exercise on energy homeostasis and metabolic disease.

